# Virtual Normalization of Physical Impairment: A Pilot Study to Evaluate Motor Learning in Presence of Physical Impairment

**DOI:** 10.3389/fnins.2017.00101

**Published:** 2017-03-14

**Authors:** Christopher Jarrett, Andrew McDaid

**Affiliations:** Department of Mechanical Engineering, Faculty of Engineering, University of AucklandAuckland, New Zealand

**Keywords:** virtual normalization, biomechatronics, robotic assessment, motor learning, rehabilitation robotics

## Abstract

Motor learning is a critical component of the rehabilitation process; however, it can be difficult to separate the fundamental causes of a learning deficit when physical impairment is a confounding factor. In this paper, a new technique is proposed to augment the residual ability of physically impaired patients with a robotic rehabilitation exoskeleton, such that motor learning can be studied independently of physical impairment. The proposed technique augments the velocity of an on-screen cursor relative to the restricted physical motion. Radial Basis Functions (RBFs) are used to both model velocity and derive a function to scale velocity as a function of workspace position. Two variations of the algorithm are presented for comparison. In a cross-over pilot study, healthy participants were recruited and subjected to a simulated impairment to constrain their motion, imposed by the cable-driven wrist exoskeleton. Participants then completed a sinusoidal tracking task, in which the algorithms were statistically shown to augment the cursor velocity in the constrained state such that it matched position-dependent velocities recorded in the healthy state. A kinematic task was then designed as a motor-learning case study where the algorithms were statistically shown to allow participants to achieve the same performance when their motion was constrained as when unconstrained. The results of the pilot study provide motivation for further research into the use of this technique, thus providing a tool with which motor-learning can be studied in neurologically impaired populations. This could be used to give physiotherapists greater insight into underlying causes of motor learning deficits, consequently facilitating and enhancing subject-specific therapy regimes.

## Introduction

A major neuroscience and clinical question is how best to invoke motor learning, a critical component of the rehabilitation process (Krakauer, 2006). It is difficult to completely understand why neurologically impaired patients cannot learn to perform certain tasks, as there are many underlying factors that contribute to motor learning deficits. Often the presence of physical impairment, such as issues with strength or range of motion (ROM), can confound results. This can make it unclear whether the inability to perform aspects of a task is physical, a neurological motor learning deficit or a combination of both.

This is illustrated in multiple domains. One example is the administration of botulinum toxin type A (BoNT-A), commonly used to reduce spasticity in children with cerebral palsy (Boyd and Graham, 1999; Wissel et al., 1999; Wallen et al., 2004; Fattal-Valevski et al., 2008, 2010; Molenaers et al., 2009, 2010; Tedroff et al., 2009). BoNT-A addresses issues due to neural spasticity and in cases where patients exhibit motion impairment after injections, it can be unclear whether this is a result of dosage, issues with the structural properties of muscle, or a fundamental incapacity for neural plasticity and motor learning. Similarly confounding issues have been reported in other studies, alluding to the uncertainty of drawing conclusions when motor learning appears poor in children with CP (van Abswoude et al., 2015). Self-exploration and problem solving are critical to the motor learning process, but these can be impacted by motor difficulties, preventing optimal learning (Valvano and Rapport, 2006).

The emergence of bio-mechatronics systems, specifically rehabilitation robotics, has provided researchers with a new suite of tools for interacting with humans and hence the ability to investigate the fundamental mechanisms of rehabilitation and motor learning (Boudreau et al., 2010; Turner et al., 2013). Robots have served as a useful research platform, igniting debate about the most efficient way to stimulate motor learning. Some authors promote the well-developed “assist-as-needed” (AAN) paradigm (Jezernik et al., 2004) while others suggest that error-augmentation (EA) is more effective (Patton et al., 2006). The most recent evidence suggests that the answer depends on a range of factors, from frequency of feedback to initial skill level (Wulf et al., 2010; Burtner et al., 2014; Marchal-Crespo et al., 2015; Fujii et al., 2016). In other words, the potential for motor learning is largely subject-dependent. Moreover, recent reviews indicate that a gap exists between current technology and true clinical needs (Cordella et al., 2016).

In order to optimize rehabilitation a therapist must better comprehend the fundamental subject-specific causes of motion deficit (Lipp and Tomassini, 2015). Recent studies have investigated the separation of neural components of torque such as spasticity, from non-neural components such as muscular stiffness (Bar-On et al., 2014, 2015; Lin et al., 2016). While this provides a valuable analysis of motion, and enables the effectiveness of various treatments to be analyzed, in these studies the cognitive ability to motor learn was not considered as a component of observed motion. Given the importance of motor learning in rehabilitation and to provide further insight into the causes of motion issues, it would be useful to evaluate motor learning independently of confounding physical impairment.

Melendez-Calderon et al. (2015) conducted a study which demonstrated motor learning could be achieved without physical motion. This built on studies focused on adaptation to environments after virtual training with visual feedback (Sarlegna et al., 2010; Melendez-Calderon et al., 2011; Rotella et al., 2014). In the case of physical impairment force production might be limited, which could affect both virtual exploration and conclusions regarding motor learning. Participants' motion could also be scaled in a purely kinematic sense and with no regard to dynamic models, as was previously used to investigate transfer of knowledge between scaling conditions (Ojakangas and Ebner, 1991; Smiley-Oyen et al., 2003; Paz et al., 2005). To the best of our knowledge no conclusive method has been developed to specifically study motor learning independently of physical impairment.

In this study, we ask whether it is possible to use a robotic exoskeleton as a tool to evaluate the motor learning capacity of an impaired individual by normalizing their physical abilities in a virtual environment and investigating their ability to learn a task in this environment. The robotic exoskeleton rehabilitation device is used to simulate a physical impairment on healthy participants and visually augment the residual motion on a display; thus “normalizing” the “impairment”. The “impairment” is manifested as a set of constraints on motion. A case study is presented, where we hypothesize that the virtual normalization of participants' abilities can be used to evaluate the cognitive ability to motor learn irrespective of physical impairment. The exoskeleton will therefore be used as a tool to diagnose sources of motion deficit and evaluate the motor learning capacity of impaired individuals, serving as a valuable aid for clinicians to create optimized, subject-specific therapy routines. This also has potential to lead to enhanced and harmonized human-robotic interaction for people with impairment. Moreover, it provides a platform for further research into motor learning in the presence of impairment.

## Materials and methods

### System architecture and model of learning

**Figure 1:**
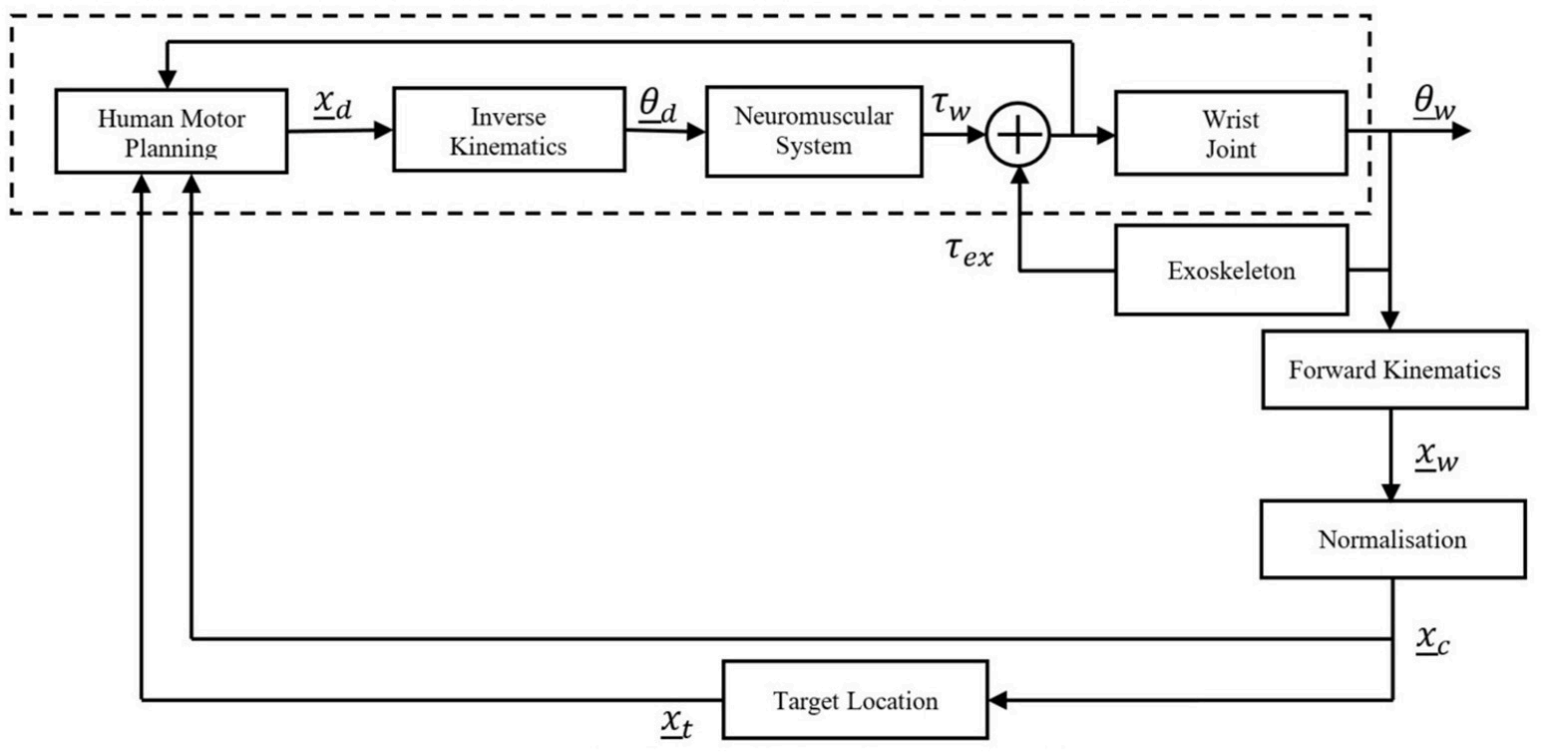
**Human-robot system architecture**.

The architecture of the entire human-exoskeleton system proposed in this paper is described in Figure 1, with the human represented by the blocks encompassed by the dashed lines.

A movement is planned, resulting a motor command which is translated into a desired Cartesian coordinate, *x*_*d*_ which is converted via inverse kinematics to a desired joint angle(s), θ_*d*_. The Neuromuscular System block encompasses the neuromuscular dynamics of the human, the output of which is a wrist torque, τ_*w*_. This human torque along with the external torque exerted on the wrist by the robotic exoskeleton, τ_*ex*_, determine the wrist kinematics. The human and robot torques are fed back internally to inform the human's motor planning. The exoskeleton torque is comprised of the resistive torque due to the constraints imposed by the simulated impairment, while the Neuromuscular System block encompasses any real impairment, which then affects the generated wrist torque.

As the wrist is coupled to the exoskeleton, the angle of the exoskeleton is equal to the angle of the wrist, θ_*w*_. This kinematic output, θ_*w*_, is converted into Cartesian coordinates, *x*_*w*_, then passed through the normalization (which is unity in the healthy state) to be presented visually on screen as feedback (in the form of the cursor) to the participant, *x*_*c*_. The behavior of the dynamic target, *x*_*t*_, is related to the normalized wrist kinematics by a simulated model, in this case a viscous damper. The target and cursor locations are viewed by the human to inform motor planning for the subsequent motion. This has been intentionally modeled as a black-box because, while it is understood the relationship between wrist movement and corresponding target movement can be learnt, literature documents multiple mechanisms by which learning occurs (Haith and Krakauer, 2013) and this is not of direct relevance to this work.

### Participants

A convenience sample of 10 participants, 8 male and 2 female, with no cognitive or physical impairments were recruited for the study. The study was approved under the guidelines of the University of Auckland Human Participants Ethics Committee (UAHPEC), reference number #013729 and written and informed consent was obtained from all participants in accordance with these guidelines. Study size was considered sufficient, given the similarity of the results in the healthy population and exploratory nature of the study, where the aim was to technically and experimentally prove the concept of virtual normalization. All participants were coincidentally dominant in the right hand.

### Exoskeleton device

**Figure 2:**
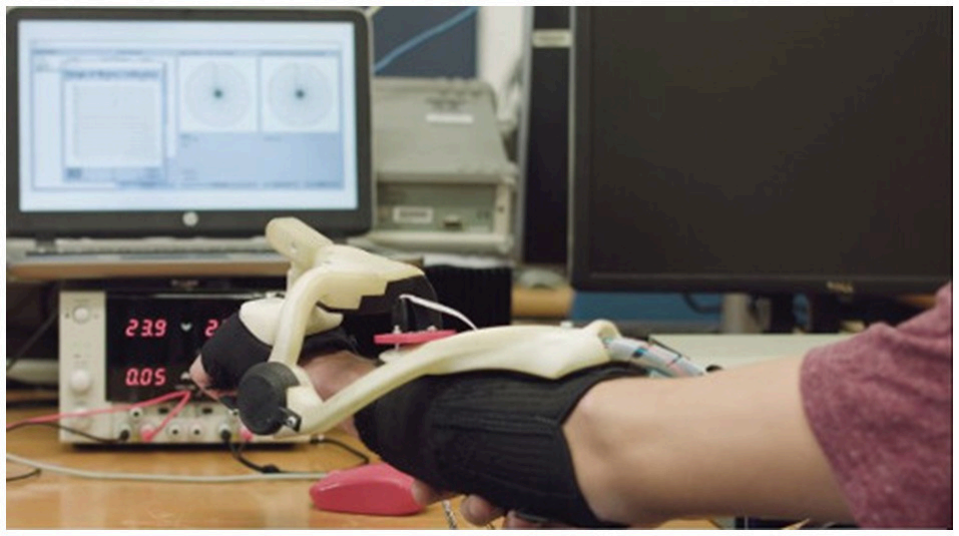
**AW-TEX Wrist Exoskeleton**.

The 3 degree-of-freedom (DOF) AW-TEX robotic wrist exoskeleton was used in this study (McDaid, 2015). Flexion/extension (FE) and radial/ulnar deviation (RU) are actively controlled. The third DOF is passive, designed to accommodate forearms of differing lengths. The exoskeleton is shown in Figure 2.

Series Elastic Actuators (SEAs) are used at the active joints and are driven by remotely located motors with Bowden cables. An inner controller based on sliding mode control regulates the relative displacement of the SEA components, thus controlling the torque in an impedance-based architecture (Jarrett and Mc Daid, 2017).

### Implementation of simulated impairment

The present study is performed with healthy participants with a simulated CP-like impairment so that we can use each person as their own control in a cross-over study. CP describes a group of disorders with a variety of phenotypic manifestations and studies have varied in their attempts to describe the “average” kinematics of people with cerebral palsy (Barroso et al., 2011; Klingels et al., 2012). In this study, the impairment is modeled with two components: (i) a simulated contraction, implemented by a linear torque spring restricting motion in the extension direction from 5°; (ii) a velocity-dependent torque, simulating restricted torque production and applied throughout the workspace. The simulated impairment is manifested as a set of “constraints,” and is mathematically described in Equations (1) and (2).

(1) $$\begin{array}{rcl} \tau_{fe} & = & \{\begin{array}{c} -sgn\left({\dot{\theta }}_{fe}\right)\left[K_{p}\left(\theta_{fe}-5\right)+K_{d}{\dot{\theta }}_{fe}+K_{v}{\dot{\theta }}_{fe}\right],\;\theta_{fe}>5^{o}\;\\ -sgn\left({\dot{\theta }}_{fe}\right)\left[K_{v}{\dot{\theta }}_{fe}\right],\;\theta_{fe}<\;5^{o} \end{array} \end{array}$$

(2) $$\begin{array}{r} \tau_{ru}=-sgn\left({\dot{\theta }}_{ru}\right)\left[K_{v}{\dot{\theta }}_{ru}\right],\;\forall \;\theta_{ru} \end{array}$$

where τ_*fe*_ and τ_*ru*_ are the torques applied to the wrist in the flexion/extension and radial/ulnar deviation axes, θ_*fe*_ and θ_*ru*_ are the flexion/extension and radial/ulnar deviation angles of the wrist, ${\dot{\theta }}_{fe}$ and ${\dot{\theta }}_{ru}$ are the angular velocities of the wrist in the flexion/extension and radial/ulnar deviation axes, *K*_*p*_ and *K*_*d*_ are the stiffness and damping of the wall that restricts the ROM; set to 2Nmdeg^−1^ and 0.1Nmsdeg^−1^ respectively, *K*_*v*_ is the gain applied to implement the velocity-based resistance; set to 0.02Nmsdeg^−1^ and *sgn* is the sign function. Operation of the exoskeleton in this mode is hereafter referred to as the “constrained” state.

### Impairment assessment

**Figure 3:**
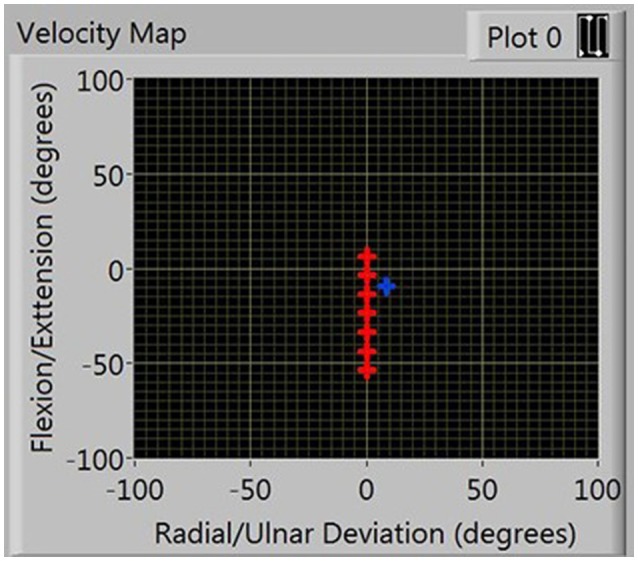
**Presentation of nodes in FE workspace during velocity calibration**.

A calibration routine was developed to evaluate the task-independent physical ability of patients in the healthy (exoskeleton in backdriven mode) and constrained states. This extracted three subject-specific measurements:

1. Self-selected neutral position. These coordinates were used as the participants' origins for the remainder of the experiments.
2. ROM, obtained by asking participants to move their wrist as far as possible in each direction.
3. Position-dependent velocity, this was recorded by asking participants to perform eight blocks of five linear movements, alternating between the flexion/extension and radial/ulnar deviation axes, (a total of four blocks per axis). Figure 3 illustrates the visual representation of the workspace shown to participants, displayed as nodes placed at 10° intervals in the workspace measured by the preceding test. They were the locations at which average velocities were measured. One minute rest periods were enforced after four blocks of movement to eliminate effects of fatigue. The routine was completed twice: first in the healthy state, then with the constraints applied and separated by a 2 min break.

### Kinematic normalization

To normalize the ability of the participants in their constrained states, two steps were required. The first is to shift the position of the cursor such that the constrained ROM centers on (0, 0). The second step is to apply a position-dependent scaling factor to the velocity of the cursor such that it matches the position-dependent velocity recorded in the healthy state. This was achieved with the aid of Gaussian Radial Basis Functions (GRBFs). GRBFs are advantageous due to their ability to describe any continuous function; and have been used in other robotic rehabilitation research to model patient torque as a function of workspace position (Wolbrecht et al., 2008; Oboe and Pilastro, 2014; Pehlivan et al., 2015). The nodes at which velocities were recorded during the calibration phase served as the locations of the RBFs.

**Figure 4:**
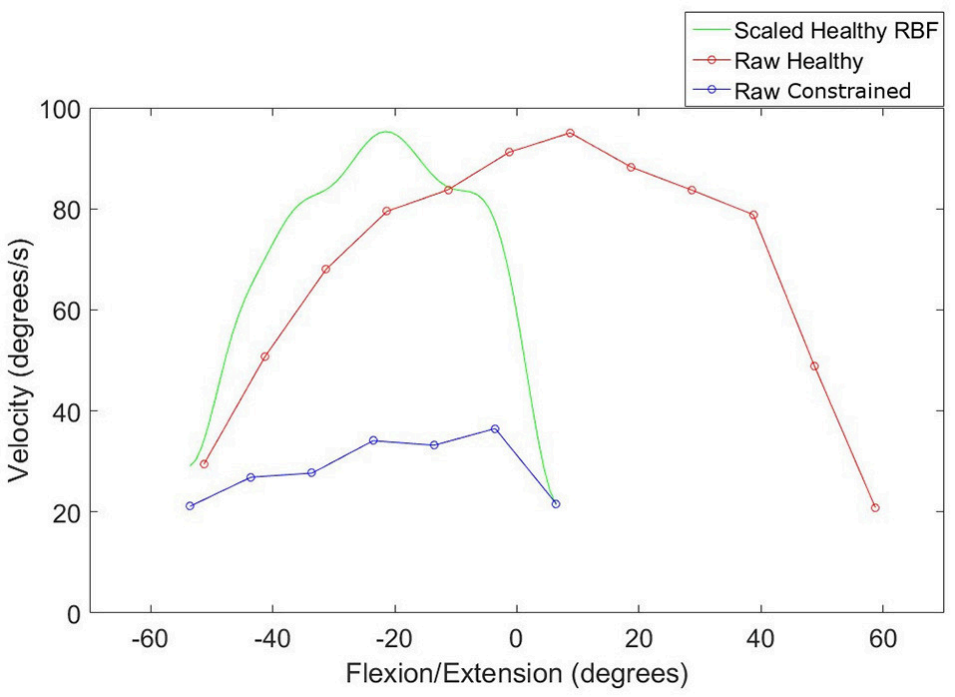
**Compression of an RBF describing healthy velocity into the constrained workspace**.

The first step was to compress and shift the node coordinates in the healthy workspace into the constrained workspace, illustrated by the green plot in Figure 4. The compressed healthy RBF was then evaluated at the nodes describing the constrained workspace. This allows the calculation of the ratio of healthy to constrained velocity at each of the constrained velocity nodes, as described by Equation (3).

(3) $$\begin{array}{r} SF_{n}=\frac{v_{hn}}{v_{in}} \end{array}$$

where *SF*_*n*_ is the scaling factor at the *n*^*th*^ constrained node, *v*_*hn*_ is the estimated healthy velocity at the *n*^*th*^ constrained node and *v*_*in*_ is the constrained velocity at the *n*^*th*^ constrained node. This creates a discrete set of scaling factors which are used to create a second RBF that normalizes velocity in the constrained workspace. The RBF allows the algorithm to capture velocity characteristics that vary with wrist position.

Equation (4) describes the RBF that was used to derive the scaling factor:

(4) $$\begin{array}{r} SF_{j}\left(\theta_{j}\right)=Ya+\text{w} \end{array}$$

where *SF*_*j*_*(*θ_*j*_*)* is the scaling factor as a function of motion at joint *j*, θ_*j*_, *Y* is the regressor matrix, composed of Gaussian basis functions as illustrated in Equation (5), *a* is a set of weightings applied to each basis function and *w* is a linear bias term applied to the output layer, calculated by multiplying an additional weighting coefficient by the current position.

(5) $$\begin{array}{r} Y=\left[\begin{array}{cc} g_{1} & 0\\ 0 & g_{n} \end{array}\right] \end{array}$$

where *n* is equivalent to the number of nodes that were obtained in the ROM test and *g*_*n*_ refers to the Gaussian radial basis function, defined in Equation (6).

(6) $$\begin{array}{r} g_{n}=e^{-\frac{d_{n}^{2}}{2\sigma^{2}}} \end{array}$$

where *e* is the exponential function, *d*_*n*_ is the distance of the cursor from the *nth* node and σ is the width of each basis function; defined as the average distance between nodes.

Equation (4) contains a linear bias term, the purpose of which is to reproduce global behavior of the RBF function (Li and Verma, 2015). In previous work in rehabilitation robotics involving RBFs, the linear bias term was omitted (Wolbrecht et al., 2008; Oboe and Pilastro, 2014; Pehlivan et al., 2015). In this research, we set the value of *w* to 0 in the RU axis, to mirror the previous studies and investigate if the linear bias term can significantly improve the normalization algorithm.

**Figure 5:**
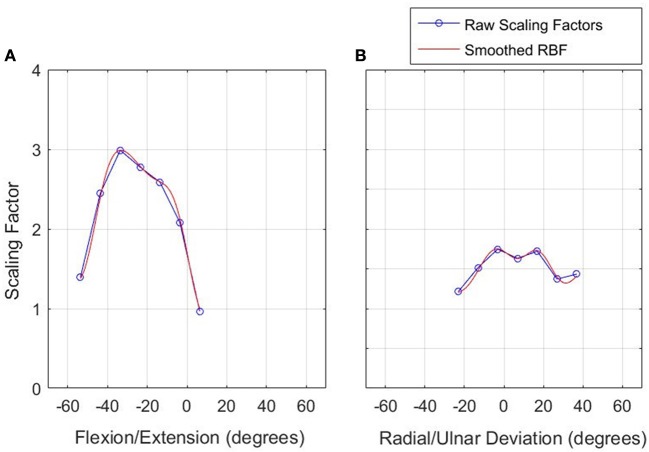
**Scaling factor function to normalize velocity in the constrained workspace**. Left: FE **(A)**, Right: RU **(B)**.

An example of the resulting scaling factor is illustrated in Figure 5 for both axes.

#### Validation of normalization algorithms

To validate the normalization, participants were subjected to the constraints with their cursor velocity normalized by the subject-specific scaling factor RBF. They were asked to try to track the moving target, which was a recording of their movement during the healthy calibration. The velocity of the cursor was recorded for comparison with the velocity of the healthy movement. Four movement blocks were performed, in the same structure as the calibration routine.

### Case study: motor learning dynamic cursor model

To evaluate the use of the algorithm in conducting kinematic motor learning experiments a task where the target has a mass-damper relationship with the cursor is implemented. The aim was to prove that when their constrained velocity was normalized, participants could perform the task to the same standard with the same amount of learning as they did in the healthy state. Eight participants, six males, and two females, continued onto this phase of the study.

#### Study design

**Figure 6:**
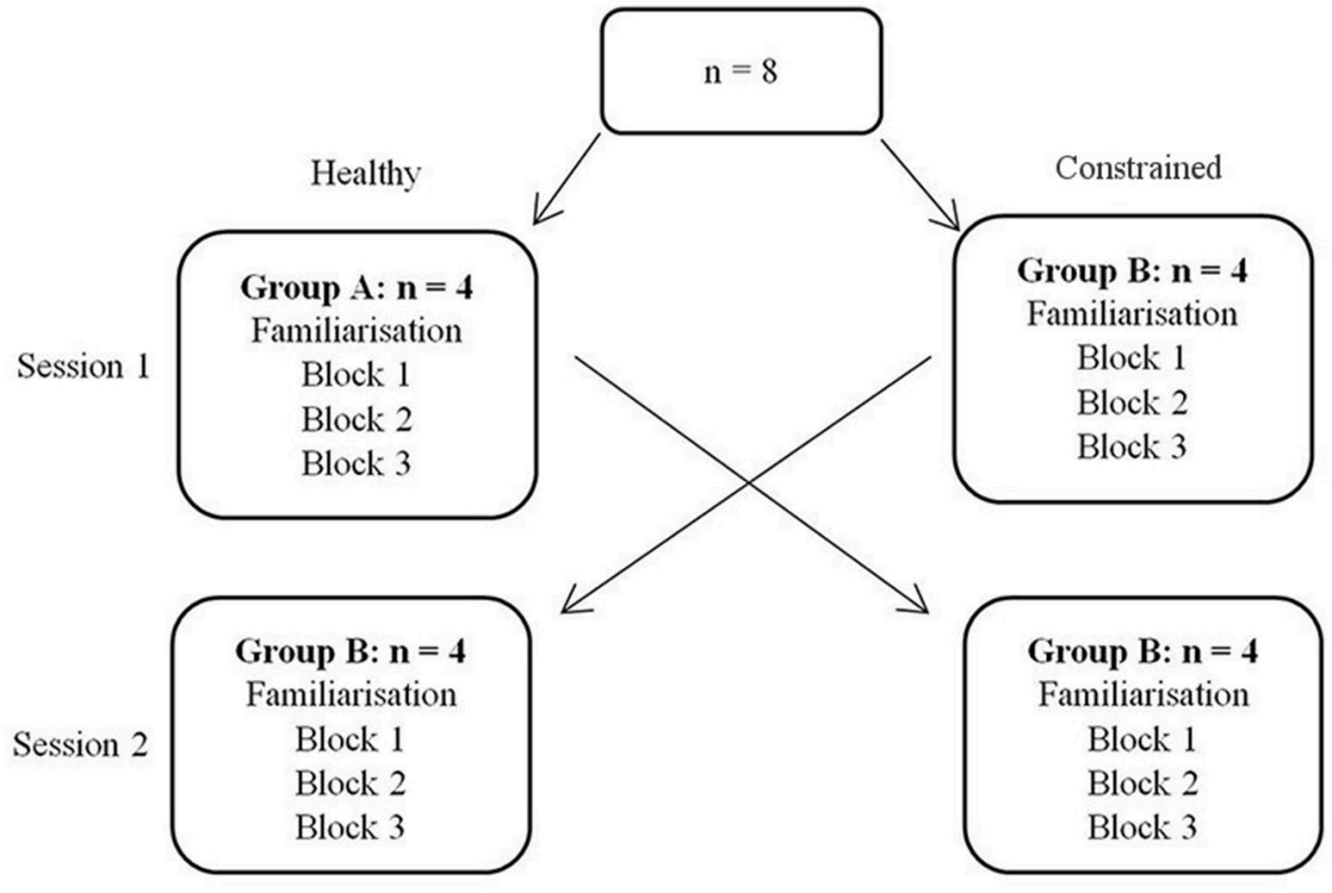
**Design of cross-over study**.

The participants were randomly separated into two groups, to conduct a cross-over study. Each group was tested for performance in the task across two sessions; each conducted in one of the healthy and constrained states. Each session consisted of a familiarization phase, followed by three blocks of movement in an acquisition phase, details below. Group A conducted the first session in the healthy state, followed by the constrained state 1 week later and vice versa for Group B. The design is illustrated in Figure 6. In the constrained state, participants' residual motion was augmented to allow them to reach the same velocity in task space as they could under “healthy” states. The 1 week break between experimental states served as a washout period, similar to comparable motor learning crossover studies (Abdollahi et al., 2014).

#### Task description

**Figure 7:**
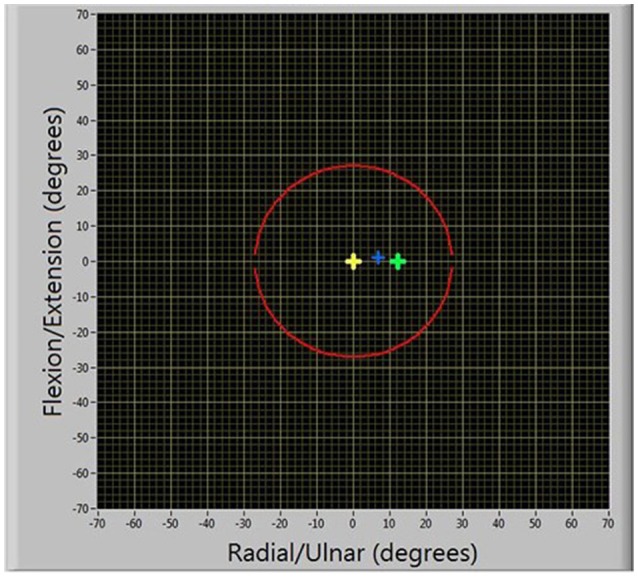
**Illustration of motor learning task**.

Participants were seated in front of a computer screen, upon which their wrist position was represented by a blue cursor and a target was illustrated in green, Figure 7. During task execution, the yellow origin was always visible. The goal of the task was to reach the target, with “success” occurring when participants had been within a 2° radius for 750 ms. Only one target appeared at a time. Targets were initially placed at a distance equal to 20% of the smallest ROM measurement across both axes. One participant's targets were placed at 25% of this distance, due to a small ROM (≈10° in the radial direction) which would have placed the initial location of the targets within the 2° threshold for target achievement. To make the task non-trivial and require some level of adaptation, the targets were programmed to be virtually connected to the participants' movement as a function of wrist velocity, according to Equation (5):

(7) $$\begin{array}{r} {\ddot{x}}_{t}=\frac{c_{t}\left({\dot{x}}_{c}-{\dot{x}}_{t}\right)}{m_{t}} \end{array}$$

where ${\ddot{x}}_{t}$ is the acceleration of the virtual target, *m*_*t*_ is the virtual mass of the target, *c*_*t*_ is the damping coefficient, ${\dot{x}}_{c}$ is the velocity of the cursor and ${\dot{x}}_{t}$ is the velocity of the target. The movement of the target is thus related to the kinematics of the participants' wrists and the difficulty can be varied. Based on pilot trials the mass and damping were set to 5 kg and 18 kgms^−1^ respectively, to ensure the task was non-trivial and requires some learning and adaptation. No external torque field was applied (except the constraints) and consequently, the task requires pure kinematic adaptation.

Participants were instructed to: “Try and catch each target by moving toward it. The target will disappear if you have been successful and turn red if you fail to achieve it.” They were further informed that a successful reach required that they were over the target for approximately 1 s and they had 10 s to reach each target. Targets were considered failed if their position exceeded the outer boundary (illustrated as the red circle in Figure 7) or if the participant had not achieved it within 10 s of it appearing.

#### Experimental procedure

##### Familiarization phase

Prior to conducting the task, participants underwent a familiarization phase, consisting of reaching 16 randomly ordered, stationary targets in both the positive and negative directions for each axis. Targets were placed at the same distance as for the main task.

##### Acquisition phase

Following familiarization, participants performed three blocks of the main task, separated by a 1–3 min break. Both targets were visited 15 times each, in a randomized order, after which the RU target was made to reappear as a stationary target, serving as a catch trial to test for after-effects. Ideally, there would have been a subsequent catch trial in the FE axis as well; however, this would have been predictable, affected by a prior catch trial in the RU axis and generally confounded results (Focke et al., 2013; Stockinger et al., 2014).

## Results

### Validation of normalization algorithm from calibration

**Figure 8:**
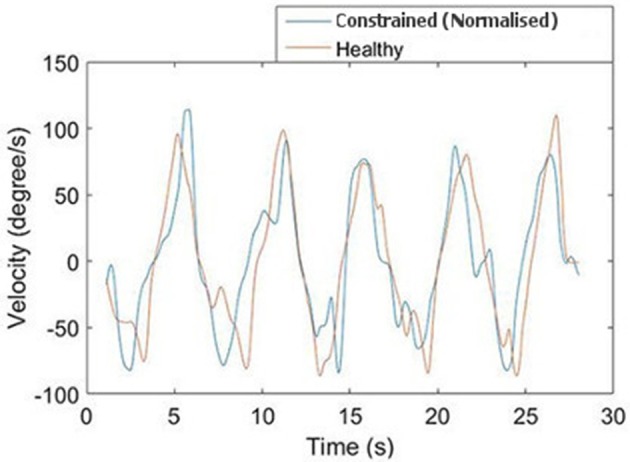
**Comparison of normalized and healthy velocities for the FE axis**.

**Figure 9:**
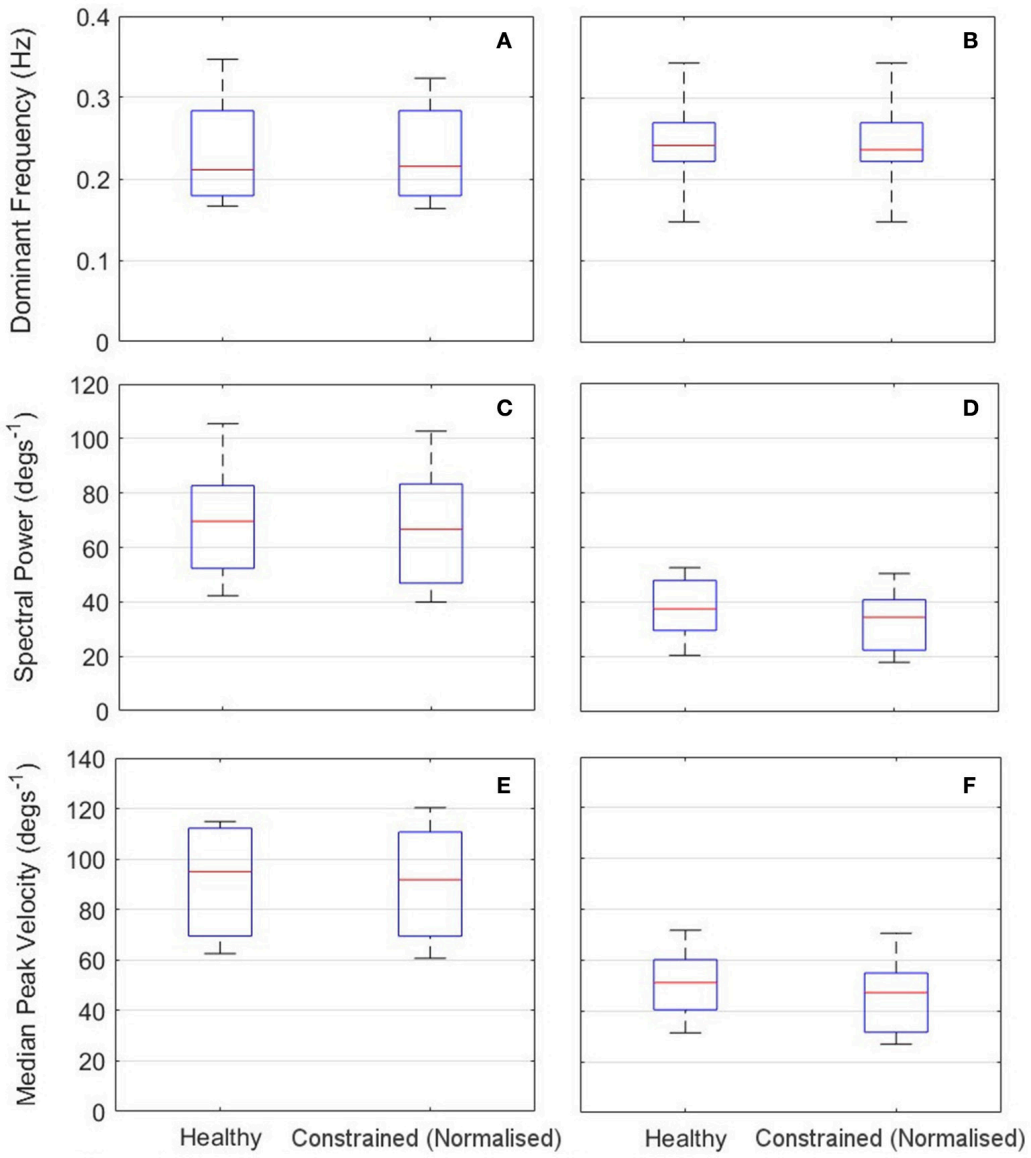
**Results of calibration validation for all participants (*n* = l0)**. Left: RU axis **(A,C,E)**; Right: FE axis **(B,D,E)**. Top: Dominant Frequency **(A,B)**; Middle: Spectral Power Component at Dominant Frequency **(C,D)**. Bottom: Median Peak Velocity **(E,F)**.

A representative response from the validation of the normalization algorithms is presented in Figure 8. Three metrics from the healthy and constrained velocity profiles are extracted and presented in Figure 9. In Tables 1, 2, the median values for all participants' metrics across the four trials from the validation are presented, in addition to the RMS error normalized as a percentage of the healthy velocity. All statistical analyses used an α level of 0.05.

**Table 1:** **Summary statistics for algorithm validation in RU axis**.

	**Dominant Frequency (Hz)**		**Power Component (degs ^−1^)**		**Peak Velocity (degs ^−1^)**		**Normalized RMS Error (%)**
	**Healthy**	**Constrained (Normalized)**	**Healthy**	**Constrained (Normalized)**	**Healthy**	**Constrained (Normalized)**	**$\frac{\text{Healthy}-\text{Constrained}}{\text{Healthy}}\times 100\%$**
Median (*n* = 10)	0.24	0.24	37.6	34.5	51.2	47.3	21.0

**Table 2:** **Summary statistics for algorithm validation in FE axis**.

	**Dominant Frequency (Hz)**		**Power Component (degs ^−1^)**		**Peak Velocity (degs ^−1^)**		**Normalized RMS Error (%)**
	**Healthy**	**Constrained (Normalized)**	**Healthy**	**Constrained (Normalized)**	**Healthy**	**Constrained (Normalized)**	**$\frac{\text{Healthy}-\text{Constrained}}{\text{Healthy}}\times 100\%$**
Median (*n* = 10)	0.21	0.22	69.6	66.6	95.0	91.8	22.1

The dominant frequency was measured by a Fast Fourier Transform (FFT). When the dominant frequency data was tested with a Wilcoxon signed rank test, there was no evidence of a difference between the healthy and constrained velocity profiles for either the RU (*p* = 0.50) or FE (*p* = 0.75) axes. Also reported are the power components measured at the dominant frequencies, a measure of the amplitude of the signal at that frequency. A Wilcoxon signed rank test on this data revealed no evidence of a statistical difference in the FE axis (*p* = 0.19) and strong evidence of a difference in the RU axis (*p* = 0.0098).

The absolute peak velocities of the constrained and healthy-target states were extracted and the median of these was used as an indicator of the peak velocity in each state. A Wilcoxon signed rank test revealed no evidence of a difference in the peak velocities for the FE axis (*p* = 0.32) but strong evidence of a difference in the RU axis velocities (*p* = 0.027), with the medians reported in Tables 1, 2. This suggests the specific normalization algorithm may have had an effect. The median RMS errors are also reported as a percentage of the maximum healthy-target velocity and were 21.0 and 22.1%for the RU and FE axes respectively.

### Motor learning task

**Figure 10:**
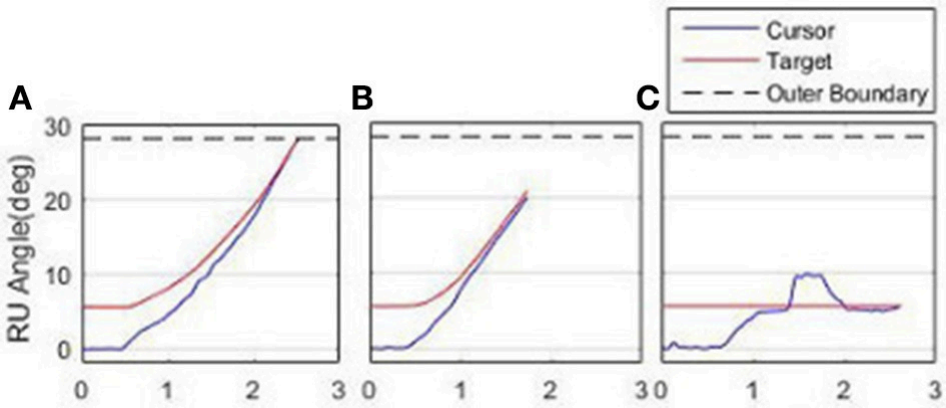
**Adaptation to achieving the RU Target. (A)** First attempt, Block l. **(B)** Final attempt, Block 3. **(C)** Catch Trial.

Participant 6's adaptation to achieving the RU Target is shown in Figure 10, comparing performance between the first RU Target in Block 1 (a), the last RU target in Block 3 (b) and the catch trial (c). In (a), the participant has failed to achieve the target before it reached the boundary. In (b), the target was successfully achieved, and (c) illustrates a clear after-effect as the participant overshot before settling on the stationary target.

Two metrics were used to measure task performance; (i) number of successful targets achieved; (ii) sum-of-squared-error (SSE) measured from the time of target appearance to success (to reflect aspects of both settling time and overshoot); in addition to magnitude of after-effects observed on the catch trial (to quantify the amount of learning).

Qualitatively observing participants indicated that they used one of two methods to complete the task. Either they would immediately move toward the target, or initially move away from the target, giving themselves more “run-up” to reach the target. Therefore, for a given participant, the after-effect is either the maximum overshoot of the target (when participants immediately moved toward the target) or the maximum initial error (when participants chose to move away from the target initially). For each participant, the metric was calculated as the median value across the three blocks.

**Figure 11:**
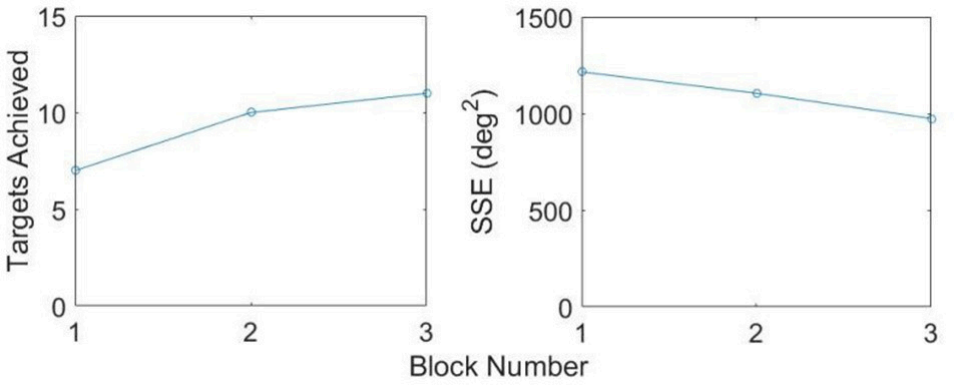
**Improvement in metrics, RU Target, Constrained**.

Figure 11 illustrates one participant's task metrics across the three blocks of trials for the RU Target in the constrained state. An increase in the number of targets achieved is accompanied by a decrease in SSE, representing an improvement in performance.

**Figure 12:**
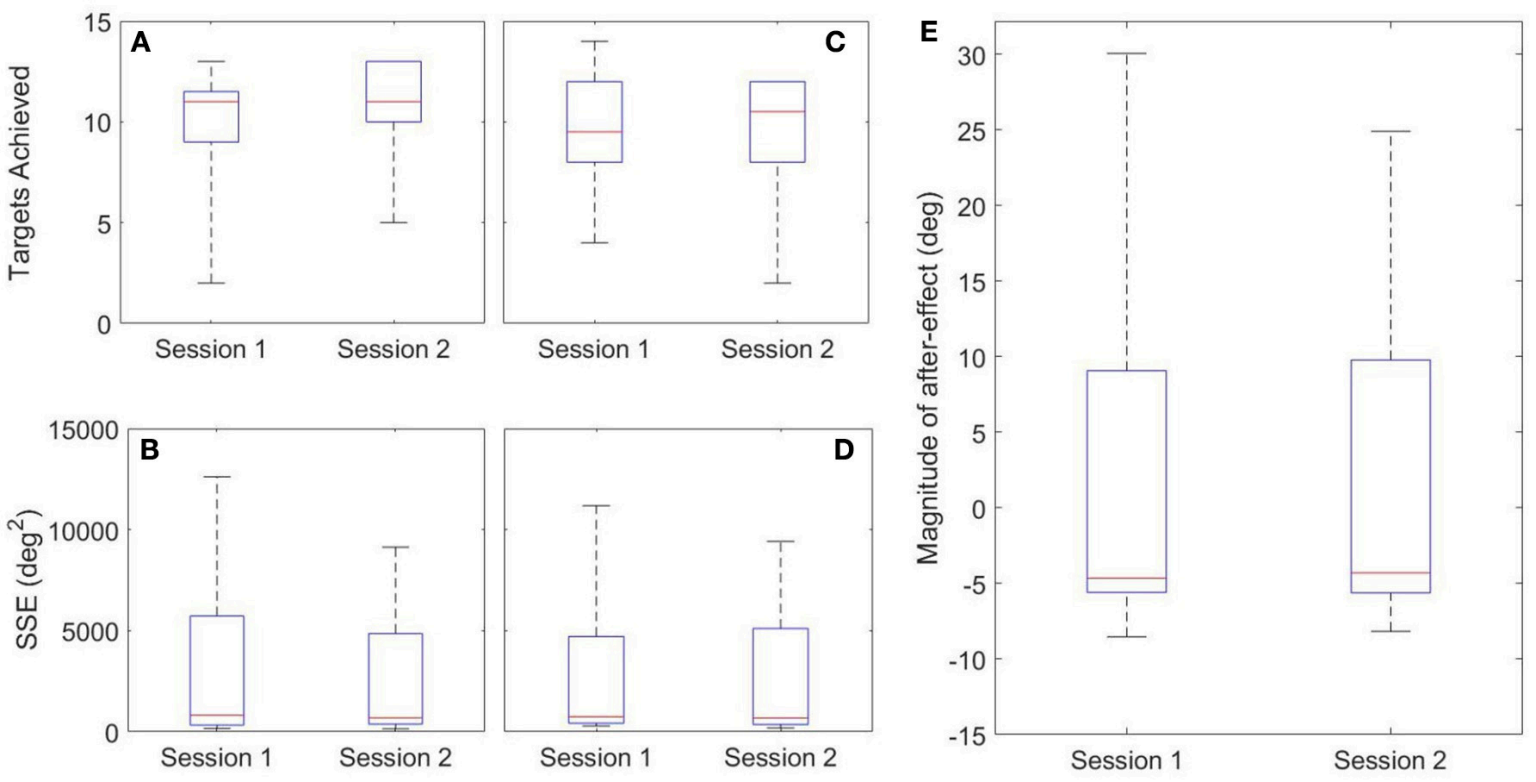
**Inter-session Results**. Left: FE Target **(A,B)**; Middle: RU Target **(C,D)**; Right: After-Effects **(E)**.

Both inter-session and inter-state analyses are presented. Inter-session analysis is performed using a Wilcoxon signed rank test, where statistical differences between sessions would imply that 1 week was insufficient time to washout learning. These results are summarized in Figure 12 and Table 3. There was no evidence of a difference between Sessions 1 and 2 for either target (FE, RU) for the number of targets achieved (*p* = 0.89, *p* = 0.94) or the SSE (*p* = 0.15, *p* = 0.20). There was also no evidence of a difference for the magnitude of after-effect on the catch trial (*p* = 0.74). The results conclude that 1 week was indeed sufficient to washout learning.

**Table 3:** **Inter-session summary statistics**.

**Metric**	**FE target**				**RU target**				**After effect magnitude**	
	**Targets achieved**		**SSE**		**Targets achieved**		**SSE**		**Distance (deg)**	
Session	1	2	1	2	1	2	1	2	1	2
Median (*n* = 8)	11	11	746.6	694.4	9.5	10.5	813.9	697.9	−4.65	−4.31

**Figure 13:**
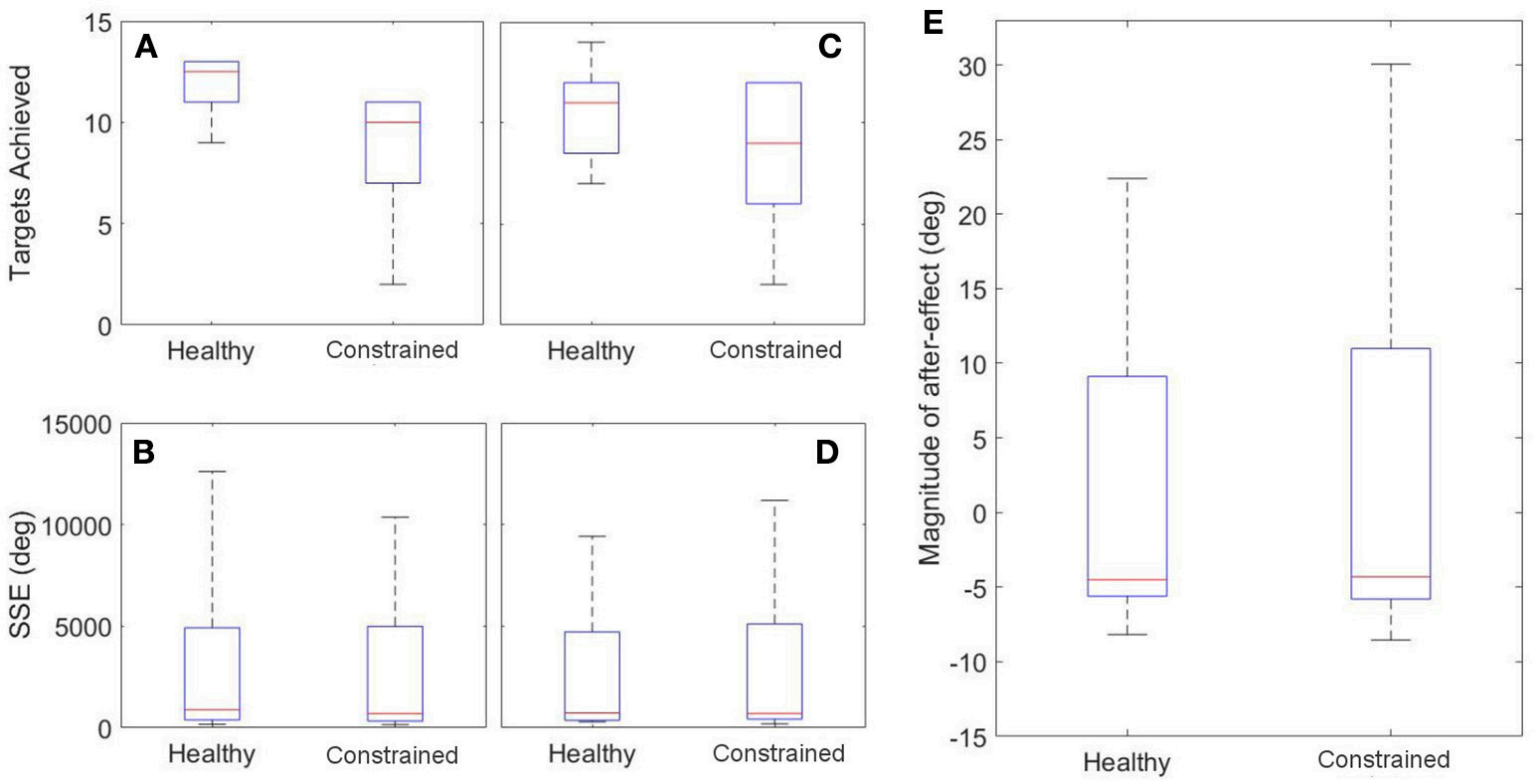
**Inter-state Results**. Left: FE Target **(A,B**); Middle: RU Target **(C,D)**; Right: After-Effects **(E)**.

Healthy and constrained states are also compared and the results summarized in Figure 13 and Table 4. Wilcoxon signed rank tests were performed and for the SSE, there was no evidence of a difference for either the FE or RU target (*p* = 0.55, *p* = 0.15). For the number of targets achieved, there was no evidence of a difference for the RU target (*p* = 0.16) but there was evidence to suggest a difference in the metric for the FE target (*p* = 0.016). For the magnitude of the after-effect on the catch trial, there was no evidence of a difference between the states (*p* = 0.74).

**Table 4:** **Inter-state summary statistics (Healthy – Constrained)**.

**Metric**	**FE target**				**RU target**				**After effect magnitude**	
	**Targets achieved**		**SSE**		**Targets achieved**		**SSE**		**Distance (deg)**	
State	Healthy	Constrained	Healthy	Constrained	Healthy	Constrained	Healthy	Constrained	Healthy	Constrained
Median (*n* = 8)	12.5	10	734.6	706.4	11	9	879.9	697.9	−4.50	−4.31

## Discussion

### Validation of normalization algorithm in calibration

Overall, the results from the calibration phase show that the algorithms were able to adequately normalize the velocity of the participants' cursor, despite their motion being constrained. For the majority of participants, the dominant frequency of motion of the normalized cursor matched that of the healthy-target motion. Where there is a difference, it is on the scale of 0.05 Hz and can be considered negligible. This is reinforced by a Wilcoxon signed rank test, suggesting no evidence of a difference in dominant frequency in either the RU or FE axes. When the power components of the dominant frequencies are considered, there is strong evidence of a difference for the RU axis, (*p* = 0.0098) and no evidence to suggest that the power components are different for the FE axis (*p* = 0.19).

The discrepancy in power components for the RU axis is not surprising, given the additional cognitive load associated with target tracking in comparison to free motion; participants constantly adjust to try and stay on the target and thus perfect tracking cannot be expected. This is reflected in the high frequency components of the graph shown in Figure 8. Moreover, the median values were similar for the constrained and healthy velocities in the RU axis (37.6 degs^−1^ and 34.5 degs^−1^), indicating that despite being unable to conclude they are statistically similar, the values are reasonably close.

Some further insight can be gained by examining the peak velocities attained in the healthy and normalized states. A Wilcoxon signed rank test on these values revealed no statistically significant differences for the FE axis, but strong evidence of a difference for the RU axis. However, the median values of healthy and constrained velocity in the RU axis are 51.2 degs^−1^ and 47.3 degs^−1^ respectively, which is a relatively small error of 7.62%. Furthermore, the boxplots in Figure 9 visually appear very similar. This suggests that the constrained velocity peaks in the RU axis were comparably close to the healthy velocity peaks.

As with the power content of the dominant frequency, some differences between peak velocities are to be expected, due to the difficulties associated with target tracking. While these metrics provide some insight into the performance of the algorithm, they are also strongly linked to individuals' cognitive abilities in target tracking. Considering the frequency components of motion, as opposed to peak velocities, offers an analysis more closely focused on the non-cognitive aspect of the motion.

The normalized RMS error was quite large, albeit similar in both the RU and FE axes, at 21.0 and 22.1% respectively. Less weight should be attached to the RMS values, however, since they are not representative indicators of the algorithms' performance due to phase lag and high-frequency motion components induced by the cognitive load of target tracking. The healthy-target was intended as a guide; thus we are not interested in matching its time-dependent position exactly. The values of the RMS error should therefore be regarded as a measure of comparing the two algorithms to each other, rather than explicitly evaluating their performance individually. From the RMS metric, it is obvious that the two algorithms have performed comparably.

Overall, we can conclude that the velocity in the constrained state was restored reasonably closely to the original healthy-target velocity by both algorithms. This justifies further exploration of this method as a tool to normalize patient physical ability in tasks requiring motor learning.

### Motor learning task

In general, the weight of evidence from this experiment suggests that both algorithms were able to normalize constrained velocities such that learning of the task was not hindered. In terms of task metrics, the only case in which there was evidence of a statistical difference was the number of times the FE target was achieved (*p* = 0.016). Further information can be gleaned by examining the median number of times the FE target was achieved in the healthy and constrained states: 12.5 and 10 respectively; implying that it was easier to reach in the healthy state.

One reason may be due to the differing algorithms used. The normalization algorithm in the FE axis was supplemented with a linear bias. Simultaneously, most participants qualitatively noted that motion with the exoskeleton felt less restrained in the FE axis. This led to greater sensitivity in the FE axis, which could have led to less consistent performance, potentially explaining the non-significant result for the FE target. Additional human biomechanical and/or exoskeleton dynamics may have contributed to differences in each axis.

One of the primary aims of this research was to create a tool with which motor learning can be studied; thus we must attempt to quantify the amount of learning that took place. The fact that task performance was similar when the algorithm was applied is promising. However, this alone does not reflect the amount of learning that occurred. Haith and Krakauer (2013) distinguish between model-based learning, requiring an internal model, and model-free learning, operating on a trial-and-error basis with no internal model formed. From a technical viewpoint, it does not matter whether the learning that occurred was model-based or model free, since both fall under the accepted definition of “motor learning,” and thus it suffices to show that the normalization allows equivalent learning across states.

Catch trials are often included in motor learning studies to test for after-effects (Abdollahi et al., 2014; Melendez-Calderon et al., 2015); and are commonly cited as evidence that an internal model was formed, since they are predictive errors (Shadmehr and Brashers-Krug, 1997; Krakauer, 2006; Haith and Krakauer, 2013). In the present study's catch trials, participants displayed substantial after-effects and no evidence of a difference was found between the healthy and constrained states for the magnitude of after-effects (*p* = 0.74). This provides evidence that the learning was the same despite the physical limitations imposed by the constraints. A limitation of the study is that the catch trial was only conducted in the RU axis and thus conclusions cannot be drawn with regards to the FE axis. This was done so as to avoid subsequent catch trials affecting each other (Focke et al., 2013; Stockinger et al., 2014). A further limitation of the catch trial analysis in this study is that two participants used different strategies to reach the target, initially pulling away from it. Therefore, the absolute values of their metrics are larger than the rest of the participants' data. While this affects the spread of the data presented, the statistical analyses remain valid, as after-effects were consistent for a given participant and paired Wilcoxon tests were used.

Haith and Krakauer (2013) also cite adaptation time as an indicator of the mechanism of motor learning. In the present study, participants required minimal adaptation time, suggesting model-based learning occurred; however, adaptation time is also influenced by task difficulty. Nevertheless, the presence of after-effects provides quantifiable evidence that learning did occur, regardless of the mechanism by which it transpired.

One limitation of the study is the simulation of physical impairment; this adds dynamics that participants must compensate for. The familiarization phases in both the impairment assessment and in the motor learning case study were designed to ensure that participants had “learnt” the constraints as much as possible before the tasks were conducted. However, in previous studies, it was found that learning of dynamic and kinematic transformations occur independently of, and in parallel to, each other (Krakauer et al., 1999). Consequently, in this study it may be that “learning” of the constraints did not interfere with learning the kinematics required to complete the task. The lack of a systematic difference in metrics between the healthy and constrained states strengthens this conclusion. However, there may be some interference in learning the transformation between physical and normalized motion with the learning of the task kinematics.

The majority of metrics used to evaluate motor learning and task performance displayed no significant differences between the states. Thus the weight of evidence concludes that the normalization allowed participants to learn and complete the task to the same standard in the constrained state as in the healthy state. This significant because it lends justification to the potential use of the normalization algorithms to conduct studies on patients with physical impairment. Physical impairment has often been cited as a confounding factor in motor learning studies, as researchers are unable to determine whether poor results are due to an inability to motor learn or because of physical barriers (Valvano and Rapport, 2006; Burtner et al., 2014; van Abswoude et al., 2015). Using normalization, therapists may be able to evaluate the cognitive ability of a patient independently of their physical impairment, alleviating the compounding factors surrounding motor learning.

It is acknowledged that the task presented here is quite specific and that it may not be considered as useful for restoring function. However, we argue that it is the concept that is important, rather than the specific task, and the concept is easily generalized to more complex tasks. Furthermore, the purpose of the normalization is not to provide restoration of physical function, but as a tool with which therapists can fairly compare individuals' (with and without impairment) inherent ability to motor learn and provide subject-specific, optimized therapy programs informed by this knowledge. This study is intended as an exploratory investigation of the concept of the normalization and future work will examine the use of this technique in patients with cerebral palsy, which will lead to further understanding of motor learning in impaired participants.

### Comparison of algorithms

The final matter to address is the comparison of each algorithm. Both algorithms were able to individually restore velocity capabilities to a decent standard. When the validation of the calibration is considered, it was the algorithm used in the FE axis that provided better results. However, it is important to consider that the only metrics where it significantly outperformed the RU algorithm were the measurement of peak velocities and the power component of the dominant frequency, which are affected by cognitive difficulties and are not considered the optimal measures of performance. Furthermore, for both algorithms, the summary statistics for all metrics were comparably close between the healthy and constrained velocity profiles.

The algorithm used in the RU axis overall provided better performance in the motor learning task, where no evidence of a difference between healthy and constrained states was found for any metric. This contrasts with the FE axis, where significantly more targets were achieved in the healthy state.

It is therefore difficult to say conclusively which algorithm was superior. The linear bias added to the algorithm in the FE axis gave no clear advantage over the standard algorithm in this application. In contrast, the algorithm in the RU axis performed sufficiently, despite the lack of a supplementing bias factor, and matches the structure of RBF used in the research that originally inspired our use of RBFs (Wolbrecht et al., 2008; Oboe and Pilastro, 2014; Pehlivan et al., 2015). Consequently, this shall be used in future development of this normalization tool.

### Limitations

The primary limitation of this study is the method of simulating physical impairment. The torques exerted by patients in the healthy and constrained cases are quite different. While this does simulate the fact that patients with impairment tend to tire more quickly than their healthy counterparts, it is possible that fatigue may be a compounding factor on the results. The focus of this preliminary study, however, was to investigate whether the kinematics of the constrained participants could be normalized and thus kinetics were not considered. In future, EMG could be used to measure torque, and further studies will focus on incorporating kinetic data into the algorithm to give a fairer normalization of the abilities of impaired individuals.

There is also difficulty associated with accurately replicating a physical impairment. In reality, these are not modeled simply. However, the purpose of this study was to show that the kinematics can be normalized in the presence of a torque field that restricts motion. In this respect, the impairment can be treated as a black box, with the only important phenomenon being that it has an impact on kinematics. The use of biomechanical models could be considered for more accurately representing actual impairments; however, this was considered to be out of scope for a preliminary study.

The pilot sample size reduces the strength of the statistical outcomes. However, as a preliminary investigation into the feasibility of the algorithm, a small sample size was considered sufficient and has gleaned significant relevant information to provide interesting results and motivate further investigation into this work.

## Conclusions

A new technique for investigating motor learning in physically impaired individuals has been proposed in an investigative pilot study focussing on wrist motion. Measurements of velocity as a function of position are used to derive subject-specific scaling factors that normalize the velocity of individuals to a “healthy” benchmark. Healthy participants were subjected to an artificial impairment that restricted both range of motion and velocity in the RU and FE axes of the wrist. The algorithms were statistically proven to restore velocity accurately. In a crossover study participants were able to complete the task with the same performance when the algorithm was applied to their constrained state as they did in the healthy state, with some evidence that motor learning had occurred. Overall, the results show promise for the further investigation of this technique in exploring motor learning in impaired individuals.

## Ethics statement

This research was supported by the Marsden Fund Council from Government funding, managed by the Royal Society of New Zealand.

## Author contributions

Both authors contributed to identifying study motivation, study design and experimental protocol. Experiments were conducted by CJ.

## Funding

This research was partially supported by the Marsden Fund Council from Government funding, managed by the Royal Society of New Zealand.

### Conflict of interest statement

The authors declare that the research was conducted in the absence of any commercial or financial relationships that could be construed as a potential conflict of interest.
